# Efficacy and Tolerability of Intramuscular Dexketoprofen in Postoperative Pain Management following Hernia Repair Surgery

**DOI:** 10.1155/2011/579038

**Published:** 2011-05-12

**Authors:** P. T. Jamdade, A. Porwal, J. V. Shinde, S. S. Erram, V. V. Kamat, P. S. Karmarkar, K. Bhagtani, S. Dhorepatil, R. Irpatgire, H. Bhagat, S. S. Kolte, P. A. Shirure

**Affiliations:** ^1^Department of Surgery, Dr. Shankarrao Chavan Government Medical College & Hospital, Nanded, Maharashtra 431601, India; ^2^Sharada Clinic, Pune, Maharashtra 411042, India; ^3^Dr. Kenekar's Noble Hospital & Research Centre, Ahmednagar, Maharashtra 414001, India; ^4^Sharada Clinic, Erram Hospital, Karad, Maharashtra 415110, India; ^5^Department of Surgery, Karnataka Institute of Medical Sciences, Hubli, Karnataka 580022, India; ^6^Shashwat Hospital, Pune, Maharashtra 411028, India; ^7^Dr. R. N. Cooper Municipal General Hospital, Vile Parle West, Mumbai, Maharashtra 400056, India; ^8^Shree Hospital, Siddharth Mansion, Nagar Road, Pune, Maharashtra 411014, India; ^9^Shri Venkatesh Hospital, Barshi Road, Latur, Maharashtra 413512, India; ^10^Criticare Hospital, JVPD Scheme, Andheri West, Mumbai, Maharashtra 400049, India; ^11^Sahyadri Speciality Hospital, Karve Road, Pune, Maharashtra 411004, India; ^12^Department of Pharmacology, Dr. Shankarrao Chavan Government Medical College & Hospital, Nanded, Maharashtra 431601, India

## Abstract

*Objective*. To evaluate the safety and efficacy of intramuscular dexketoprofen for postoperative pain in patients undergoing hernia surgery. *Methodology*. Total 202 patients received single intramuscular injection of dexketoprofen 50 mg or diclofenac 50 mg postoperatively. The pain intensity (PI) was self-evaluated by patients on VAS at baseline 1, 2, 4, 6, and 8 hours. The efficacy parameters were number of responders, difference in PI (PID) at 8 hours, sum of analogue of pain intensity differences (SAPID), and onset and duration of analgesia. Tolerability assessment was done by global evaluation and adverse events in each group. *Results*. Dexketoprofen showed superior efficacy in terms of number of responders (*P* = .007), PID at 8 hours (*P* = .02), and SAPID_ 0–8 hours_ (*P* < .0001). It also showed faster onset of action (42 minutes) and longer duration of action (6.5 hours). The adverse events were comparable in both groups. *Conclusion*. Single dose of dexketoprofen trometamol 50 mg given intramuscularly provided faster, better, and longer duration of analgesia in postoperative patients of hernia repair surgery than diclofenac 50 mg, with comparable safety.

## 1. Introduction

Pain is a common surgery-related reason for unexpected hospital admissions. Inadequate pain control can result not only into harmful physiological consequences like nausea, vomiting, increased rate of venous thromboembolism, and delayed wound healing, but also psychological ill-effects like anxiety, anger, depression, and reduced patient satisfaction [1]. 

Nonsteroidal anti-inflammatory drugs (NSAIDs) and opioids are considered effective analgesics for postoperative pain control [2]. NSAIDs alone provide adequate pain relief after minor surgery and have been shown to reduce opioid requirement following major surgeries [3].

Dexketoprofen or (S)-(+)-ketoprofen is several times more potent than its racemate, ketoprofen. The levorotatory isomer is 100 times less potent as a cyclooxygenase-2 inhibitor than dexketoprofen, however, may contribute to the ulcerogenic effect of the drug [4]. 

Dexketoprofen has been shown to possess comparable efficacy and better tolerability over ketoprofen in several clinical studies [5]. Earlier published studies have shown the efficacy and safety of parenteral dexketoprofen in postoperative pain following major orthopedic surgery [6], renal colic [7], and acute low back pain [8]. The present study evaluates the analgesic efficacy and tolerability of single dose of intramuscular dexketoprofen trometamol in postoperative patients of hernia surgery in comparison with intramuscular diclofenac.

## 2. Materials and Methods

### 2.1. Approvals

Prior approval was obtained from Drug Controller General of India (DCGI) and appropriate ethics committees. The study was conducted in accordance with Good Clinical Practice guidelines (issued by Central Drugs Standard Control Organization, Government of India) and according to the Declaration of Helsinki. The trial was registered at the Clinical Trials Registry—India (http://www.ctri.in/). Written and informed consents were obtained from all patients before their enrollment.

### 2.2. Patients

A total of 202 patients were recruited in this trial. The study population comprised of patients of either sex with age between 18–65 years undergoing hernia surgery repair (inguinal/incisional/umbilical/congenital/others). The major exclusion criteria were: known hypersensitivity to the study medications, complication during or after surgical procedure, active or suspected gastrointestinal ulcer or chronic dyspepsia or gastrointestinal bleeding, inflammatory bowel disease, history of bronchial asthma; major organ dysfunction, hemorrhagic diathesis, and other coagulation disorders. General contraindications to the use of NSAIDs as well as concomitant treatment with any anti-inflammatory or other therapy known to affect the study outcome were also considered criteria for exclusion.

### 2.3. Study Design and Medications

This was multicentric, open-label, randomized, comparative clinical trial conducted over 11 centers across India. The randomization was carried out online at http://www.randomization.com/ in the blocks of ten. 

Eligible patients were randomly assigned to receive either single intramuscular dose of dexketoprofen trometamol 50 mg (Emcure Pharmaceuticals Ltd, India) or diclofenac sodium 50 mg (from commercial source) postoperatively, when patient first complained of pain. The drugs were administered slow intramuscularly after a washout period of at least 45 minutes of intravenous and 4 hours of intramuscular analgesic, if given intraoperatively. Any medication which was considered necessary for the patient was given at the discretion of investigator provided it did not interfere with the study drug evaluation.

### 2.4. Efficacy Variables

The pain intensity (PI) was self-evaluated by the patients on visual analogue scale (VAS) (0: no pain, 100: maximum pain) at intervals of 1, 2, 4, 6, and 8 hours after the injection. Patient showing at least 50% improvement was termed as a responder. Proportion of responders in each group (responder rate) at the end of 8 hours was taken as primary efficacy variable. 

The secondary efficacy variables included time of onset of significant decrease in VAS score, pain intensity difference (PID) at the end of 8 hours, and sum analogue of pain intensity difference (SAPID) over 8 hours. 

PID was calculated for each observation by subtracting the present PI from the baseline value. SAPID_0–8 hours_ was calculated as the weighted sum of the PIDs obtained from *t* = +1 hour (hr) to *t* = 8 hours (hr) on VAS using the following equation [8]:

(1) $$\begin{array}{cc} & \text{SAPID}\\ & =\sum \left[{\text{PID}}_{t}\times \text{time}\;\left(\text{hr}\right)\;\;\text{elapsed}\;\text{since}\;\text{previous}\;\text{observation}\right]. \end{array}$$

In addition to this, patient's assessment of onset and duration of analgesia as well as patients' and physicians' global assessment of efficacy were done.

### 2.5. Safety Variables

Laboratory investigations were done at baseline as well as at discharge or on 3rd postoperative day whichever was earlier. Tolerability was assessed by recording patient's global assessment about the tolerability of the drug and percent of the patients experiencing any drug-related adverse events.

### 2.6. Statistical Analysis

The sample size was calculated by using GraphPad StatMate version 2.00 for Windows, GraphPad Software. A sample size of 90 in each group had 80% power to detect an increase of 20% responder rate with a significance level (alpha) of 0.05 (two tailed). Fischer's exact test was applied to observe, if there are significant differences between the responder rates. The decrease in PI (VAS score), PID, SAPID, onset of analgesia, and duration of analgesia were calculated (Mean ± SD) for each group and compared between the groups by using unpaired *t* test. The within-group comparison of VAS scores was done using paired *t* test. Tolerability was assessed by evaluating the percentage of patients reporting side effect. Analysis of adverse events and global assessment of safety and efficacy was done using Fisher's exact test. For all statistical tests, a “*P*” value of less than  .05 was considered significant.

## 3. Results

Total 204 patients were screened and enrolled in the study. Two patients did not continue with the study as they did not report for the surgery. So, total 202 patients received the medications and completed the study; of whom 99 received dexketoprofen and 103 received diclofenac. Demographic parameters and baseline pain scores between the two groups are shown in Table 1.

**Figure 1:**
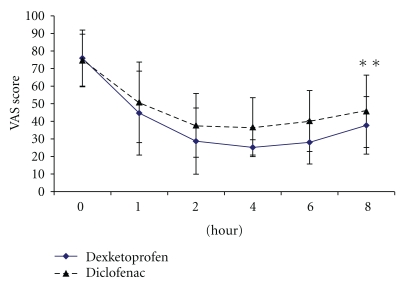
Change in pain intensity over 8 hours after dexketoprofen and diclofenac injection. **P value between the group at 8 hours =  .003 calculated using unpaired *t* test.

The responder rate in the dexketoprofen group (55.56%) was statistically superior to that in diclofenac group (35.92%) (*P* = .007). As shown in Figure 1, the VAS scores differed significantly between baseline and 8th hour in each group (*P* < .0001) indicating the efficacy of each medication. The dexketoprofen group showed significantly greater reduction in mean VAS score compared to diclofenac group (*P* = .003). 

As shown in Table 2, the comparison of PID and SAPID_0–8 hours_ after 8 hours of injection showed significantly higher values for the dexketoprofen group than diclofenac group (*P* = .02 and *P* < .0001 resp.).

The onset of analgesia for dexketoprofen was significantly faster than diclofenac by a mean of approximately 18 minutes (*P* < .0001). The duration of analgesia for dexketoprofen was also significantly greater than diclofenac with the mean difference of about 50 minutes (*P* = .003). 

The incidence of adverse events in dexketoprofen group (26.26%) was comparable to that in diclofenac group (20.38%). Pain at the injection site was the most common adverse reaction with the incidence being 25.25% and 20.38% in dexketoprofen and diclofenac groups, respectively, (*P* = .41). In addition to this, mild itching was observed in one patient receiving dexketoprofen.

**Figure 2:**
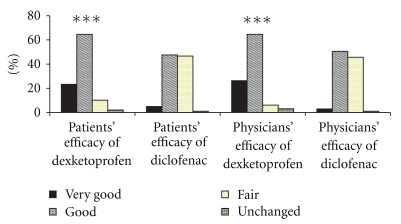
Patients' and physicians' global assessment of efficacy ***P < .0001 with Fisher's exact test for proportions between (very good + good) versus (fair + unchanged).

**Figure 3:**
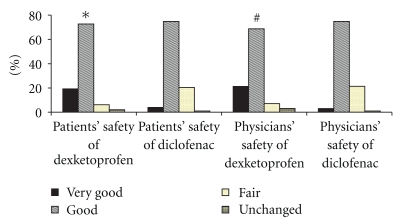
Patients' and physicians' global assessment of safty *P = .0097 and ^#^ *P* = .02 with Fisher's exact test for proportions between (very good + good) versus (fair + unchanged).

As shown in Figure 2, both patients' and physicians' overall assessment of efficacy was in favor of dexketoprofen with significantly greater number of patients showing very good and good efficacy as compared to diclofenac. Similarly, the patients' as well as physicians' assessment of safety was significantly better for dexketoprofen group as compared to diclofenac group as depicted in Figure 3.

## 4. Discussion

The goal of postoperative pain management is to relieve pain while keeping side effects to minimum. NSAIDs are effective analgesics without undesirable side effects of opioids like sedation, respiratory depression, nausea and vomiting, and so forth. They inhibit the synthesis of prostaglandins which are responsible for pain, fever, and vasodilatation in response to trauma. These drugs are particularly useful in managing the pain associated with minimally invasive surgery like hernia repair [1]. 

As compared to several other NSAIDs, ketoprofen has relatively higher analgesic than anti-inflammatory activity favoring its use in acute pain conditions [5]. Dexketoprofen, the pure S(+) enantiomer, appears to be responsible for the major analgesic efficacy [4]. Development of dexketoprofen as a chirally pure drug entity offers certain advantages like requirement of low doses, reduction in the metabolic load, avoidance of interactions, and adverse effects due to R(−) enantiomer [9]. Dexketoprofen has been developed as a trometamol salt with the aim of providing rapid onset of action, reduced gastric irritation, and improved tolerability. Increased water solubility of trometamol salt is partly responsible for these properties [4, 10]. Also, the trometamol salt undergoes rapid hydrolysis into the plasma releasing the lipophilic dexketoprofen, enabling its entrance into the CNS. As a consequence, the side effects are claimed to be lower at the therapeutic doses [11]. 

In the present study, single dose of dexketoprofen 50 mg given intramuscularly could provide adequate pain relief in postoperative pain which was significantly better than the comparator, diclofenac 50 mg, as reflected from increased number of responders. The higher values of PID and SAPID at the end of 8 hours indicates longer duration of action of dexketoprofen trometamol. The onset of action of dexketoprofen was also found to be faster than diclofenac which confirms the results of earlier studies. 

The overall adverse effect profile of dexketoprofen appeared to be no different from diclofenac with similar incidence of adverse events. However, the subjective assessment of tolerability and efficacy by patients and physicians distinctly reflected the superiority of dexketoprofen over diclofenac.

The present study confirms the findings of earlier clinical studies on oral as well as parenteral dexketoprofen that have demonstrated faster onset of action and good safety and efficacy of dexketoprofen trometamol. The present study has a limitation that it was an open-label study. The dose of diclofenac was kept as 50 mg in order to compare equivalent milligram for milligram efficacy of dexketoprofen versus diclofenac. The 50 mg dose diclofenac has also been used in earlier studies with dexketoprofen [12].

## 5. Conclusion

Single dose of dexketoprofen trometamol 50 mg given intramuscularly provided faster, better, and longer duration of analgesia in postoperative patients of hernia repair surgery than diclofenac 50 mg, with comparable safety.

## Figures and Tables

**Table 1:** Demographic and baseline data.

Variable	Dexketoprofen	Diclofenac	*P* value
No. of Patients	99	103	—
Age (Mean ± SD)	46.98 ± 13.51	42.40 ± 14.50	.02
Weight (Mean ± SD)	60.87 ± 9.75	62.20 ± 9.50	.33
Male : Female	90 : 9	90 : 13	.5
Baseline VAS score (Mean ± SD)	75.93 ± 14.96	74.61 ± 15.97	.54

**Table 2:** Efficacy parameters of dexketoprofen and diclofenac.

Variable	Dexketoprofen	Diclofenac	*P* value
Responder rate (%)	55.56%	35.92%	.007
VAS score at 8th hour (Mean ± SD)	37.67 ± 20.56	45.72 ± 16.34	.003
PID at 8th hour (Mean ± SD)	38.26 ± 36.66	28.62 ± 20.93	.02
SAPID (Mean ± SD)	352.42 ± 159.65	260.82 ± 115.34	<.0001
Onset of analgesia (min) (Mean ± SD)	42.27 ± 18.60	60.05 ± 28.87	<.0001
Duration of analgesia (min) (Mean ± SD)	391.52 ± 133.98	341.65 ± 100.23	.003

VAS: visual Analogue scale; PID: pain Intensity Difference; SAPID: sum of Analogue pain intensity differences.
